# 670. Clinical Characteristics and Outcomes in Patients with Healthcare Facility-Onset *Clostridiodes difficile* Infections

**DOI:** 10.1093/ofid/ofad500.732

**Published:** 2023-11-27

**Authors:** Seema Joshi, Rija B R Alvi, Clare Shanahan, Abigail Ruby, Eman Chami, Geehan Suleyman

**Affiliations:** Henry Ford Hospital, Detroit, Michigan; Henry Ford Hospital, Detroit, Michigan; Henry Ford health, Detroit, Michigan; Henry Ford Health, Detroit, Michigan; Henry Ford Hospital, Detroit, Michigan; Henry Ford Health, Detroit, Michigan

## Abstract

**Background:**

Healthcare facility-onset (HCFO) *Clostridiodes difficile* infection (CDI) is the most common hospital-acquired infection. Although risk factors associated with CDI have been described, characterization and outcome of HFCO-CDI are limited.

**Methods:**

This was a retrospective observational study comparing disease severity among adult patients with HCFO-CDI from January 1, 2020, to December 31, 2022, at an 877-bed tertiary care hospital in Detroit. Patients were identified using National Healthcare Safety Network (NHSN) definition. CDI was classified as nonsevere, severe, or fulminant. Severe disease was defined as having white blood cell (WBC) count ≥ 15,000 cells/mm^3^ or acute kidney injury (AKI) defined as increase in creatinine of ≥ 0.3mg/dL within 48 hours of diagnosis. Fulminant disease included patients with ileus or toxic megacolon, or need for colectomy, intensive care unit or vasopressors. Risk factors, treatment and outcomes were evaluated.

**Results:**

98 patients were diagnosed with HFCO CDI during the study period (Table 1); 37 (38%) were non-severe, 47 (38%) severe and 14 (24%) fulminant. Median age was 66 years, 50% were female and 45% white. Almost half were immune suppressed; 5% had prior CDI. Most patients (88%) were exposed to antibiotics (abx) prior to CDI with no difference between the groups (p=0.427); 61% received cephalosporins. Cirrhosis was more common among patients with fulminant disease (p=0.048) and receipt of chemotherapy was associated with severe and/or fulminant disease cases (p=0.049). AKI (p< 0.001), fever (p=0.030), and WBC >25,000 or < 2,000 cells/mm^3^ (p< 0.001) were more prevalent among patients with fulminant CDI; combination or alternative therapy was more common among fulminant cases (p< 0.001). Most were eligible, but only 6% received bezlotoxumab (BZX). Although outcomes were not significantly different between the groups, length of stay was longer and refractory disease and recurrence were more common in severe/fulminant CDI.

**Figure:**
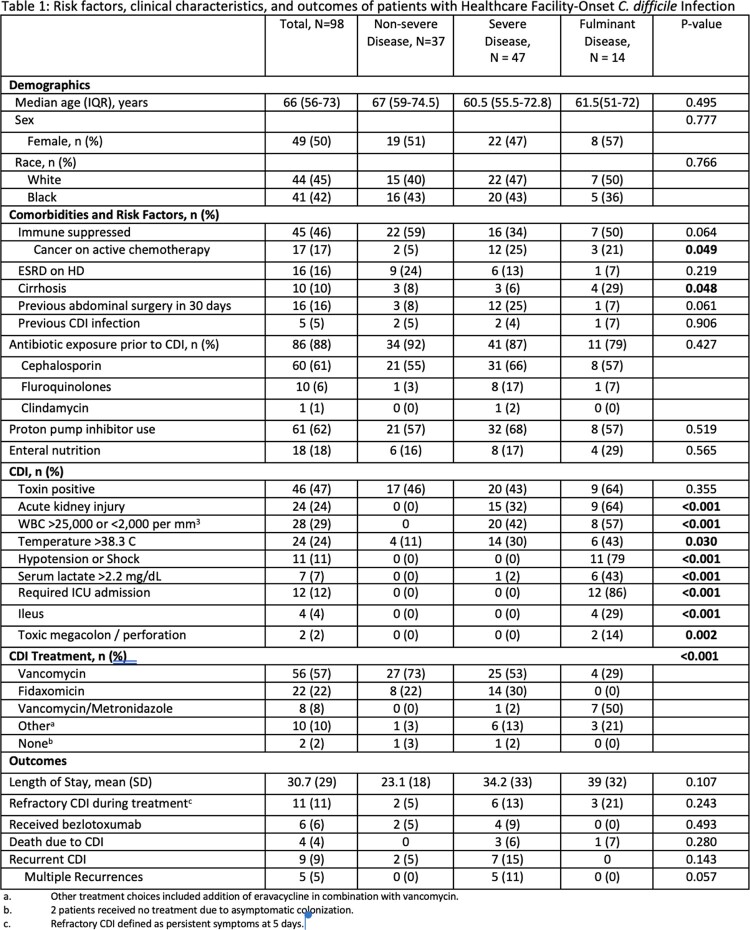

**Conclusion:**

In our HCFO-CDI cohort, most patients were exposed to abx, and cirrhosis and chemotherapy were associated with more severe CDI. Efforts should focus on appropriate abx utilization and increasing use of BZX to reduce burden of CDI and risk of recurrence and readmission.

**Disclosures:**

**All Authors**: No reported disclosures

